# Promising Directions in Atherosclerosis Treatment Based on Epigenetic Regulation Using MicroRNAs and Long Noncoding RNAs

**DOI:** 10.3390/biom9060226

**Published:** 2019-06-11

**Authors:** Daria Skuratovskaia, Maria Vulf, Aleksandra Komar, Elena Kirienkova, Larisa Litvinova

**Affiliations:** Laboratory of Immunology and Cell Biotechnology, Immanuel Kant Baltic Federal University, 236016 Kaliningrad, Russia; mary-jean@yandex.ru (M.V.); alexandkomar@gmail.com (A.K.); elenamed@list.ru (E.K.); larisalitvinova@yandex.ru (L.L.)

**Keywords:** atherosclerosis, miRNA, siRNA, lncRNA, epigenetic

## Abstract

Atherosclerosis is one of the leading causes of mortality from cardiovascular disease (CVD) and is a chronic inflammatory disease of the middle and large arteries caused by a disruption of lipid metabolism. Noncoding RNA (ncRNA), including microRNA (miRNA), small interfering RNA (siRNA) and long noncoding RNA (lncRNA), was investigated for the treatment of atherosclerosis. Regulation of the expression of noncoding RNA targets the constituent element of the pathogenesis of atherosclerosis. Currently, miRNA therapy commonly employs miRNA antagonists and mimic compounds. In this review, attention is focused on approaches to correcting molecular disorders based on the genetic regulation of the transcription of key genes responsible for the development of atherosclerosis. Promising technologies were considered for the treatment of atherosclerosis, and examples are given for technologies that have been shown to be effective in clinical trials.

## 1. Introduction

Cardiovascular diseases (CVDs) are the leading cause of death worldwide. These diseases take the lives of 17.9 million people annually, which is 31% of all deaths in the world [1]. Atherosclerosis is one of the leading causes of mortality from CVD and is a chronic inflammatory disease of the middle and large arteries caused by a disruption of lipid metabolism. The formation of atherosclerosis is the result of functional disorders in the arterial wall [2]. The accumulation of low-density lipoprotein (LDL) leads to the activation of endothelial cells. This process causes an inflammatory response, promoting the recruitment of monocytes to the intima, where they differentiate into macrophages and absorb modified lipoproteins and turn into foamy cells [3]. Atherosclerotic foci are characterized by a fibrous cap that covers the necrotic nucleus rich in lipids and are characterized by an accumulation of white blood cells in the marginal areas [3]. The latter destabilize plaque by modulating the phenotype of endothelial cells and proteolytic degradation of the components of the extracellular matrix. Such unstable lesions can rupture and lead to myocardial infarction or stroke.

Thousands of regulatory noncoding RNAs (ncRNAs) have been found in the human genome, including small noncoding RNA: microRNAs (miRNAs) and small interfering RNAs (siRNAs), and various classes of long ncRNAs (lncRNAs) [4]. It is now clear that these RNAs play a critical role as regulators of transcription and post-transcription and as directing chromatin-modifying complexes [4].

The use of ncRNA is a promising approach in the treatment of atherosclerosis. MiRNAs are small noncoding double-stranded molecules measuring 21–23 nucleotides in length that are involved in the posttranscriptional regulation of gene expression [2,5]. These RNAs consist of an inactive sense strand, an equal sequence of the target mRNA, and an antisense active strand [5]. MiRNAs occur endogenously, folding back on themselves and form short hairpins [5], siRNAs are either introduced exogenously into cells or are produced endogenously from miRNAs [5]. The mechanism of action of many siRNAs is similar to the action of siRNAs; however, siRNAs act exclusively on one gene, while miRNAs have multiple targets [5]. MiRNAs and siRNAs contribute to gene silencing, degradation, prevent-translation, which makes them a useful tool for the treatment of a number of diseases.

LncRNAs have recently become important regulators of gene expression, control of the nucleus architecture and modulation of mRNA stability, translation and post-translational modifications in the cytoplasm [6]. LncRNA of all kinds are involved in a number of disease development processes, including atherosclerosis, but the knowledge of the mechanisms by which they operate is still limited. At the same time, a small number of well-studied lncRNAs gave us important clues about the biology of these molecules and the potential of their use for therapeutic purposes.

In this review, attention is focused on approaches to correcting molecular disorders based on the genetic regulation of transcription of key genes responsible for the development of atherosclerosis. Promising technologies were considered for the treatment of atherosclerosis, and examples of those that have already been shown to be effective in clinical trials are given.

## 2. Therapeutic Approaches Based on the Use of miRNAs and siRNAs Aimed at the Disruption of Lipid Metabolism

Atherosclerosis is considered a chronic disease characterized by the activation of innate and adaptive immunity [7]. The main cause of the development of atherosclerosis is a disruption of lipid metabolism associated with dysfunction of apolipoprotein B (apoB) [7,8]. The latter is a cofactor of enzymes, receptor ligands and lipid transporters that regulates lipoprotein metabolism and tissue uptake [9,10]. Numerous studies have demonstrated the involvement of LDL and apoB, for example, very low-density lipoproteins (VLDL) and their residues in the formation of atherosclerosis [10,11].

The key event in the initiation of atherosclerotic lesions is the retention and accumulation of cholesterol-rich apoB-containing lipoproteins in the intima of the arteries in places that contribute to the formation of plaques [12]. Low-density lipoprotein and other apoB-containing lipoproteins with diameters of less than 70 nm freely pass through the arterial intima [11]. However, when the level of LDL-C is exceeded, the probability of internal retention of LDL increases, leading to the initiation and progression of atherosclerotic disease [9,11].

Low-density lipoprotein particles compose approximately 90% of the entire circulating apoB-containing fraction of lipoproteins in the fasting blood in most people [11]. However, in clinical practice, plasma LDL is usually not measured directly but is measured by the concentration of its LDL cholesterol, an indicator of the total amount of cholesterol contained in LDL particles [11]. Thus, the level of LDL in plasma has become an important parameter for assessing the risk of CVDs and obtaining a therapeutic effect [13].

Today, there are many highly effective substances that reduce the level of LDL. Currently, new long-acting drugs are being developed to reduce lipid levels [14]. Thus, the role of miRNA in the regulation of gene expression associated with the functioning of the endothelium and the development of atherosclerosis is being actively studied [15]. Large-scale global studies have identified numerous miRNAs, which are important regulators of lipid metabolism [16]. The mechanisms of specific activation of atheroprotective miRNAs and miRNA expression for proatherogenic miRNAs are currently being actively studied (Table 1). MiRNAs can regulate several genes at once in several molecular paths. Due to miRNAs’ pleiotropic mechanism of action [17], manipulating a single miRNA can potentially cause a therapeutic effect in different cells and tissues.

The miRNAs and siRNAs of the RNA-induced silencing complex (RISC) are able to bind to a complementary site in the 3′ untranslated region of the target messenger RNA (mRNA), which leads to posttranscriptional endonuclease cutting of the complementary target mRNA or its exonuclease degradation or translational suppression [18]. MicroRNA can also induce the deadenylation and decapping of mRNA [19]. As soon as the siRNA and miRNA is introduced into the cytoplasm of the cell, the active antisense chain is incorporated into the RISC complex [20]. This leads to the silencing of genes using two different mechanisms, depending on the degree of base pairing between the antisense chain and the mRNA target. With siRNA, complete homology between antisense and target mRNA leads to site-specific cleavage and degradation of mRNA [19,20]. On the contrary, partial sequence identity between the miRNA active chain and its target leads to inhibition of translation, decapitation and subsequent degradation of mRNA [20].

### 2.1. Therapeutic Approaches Based on the Use of miRNAs

**Figure 1:**
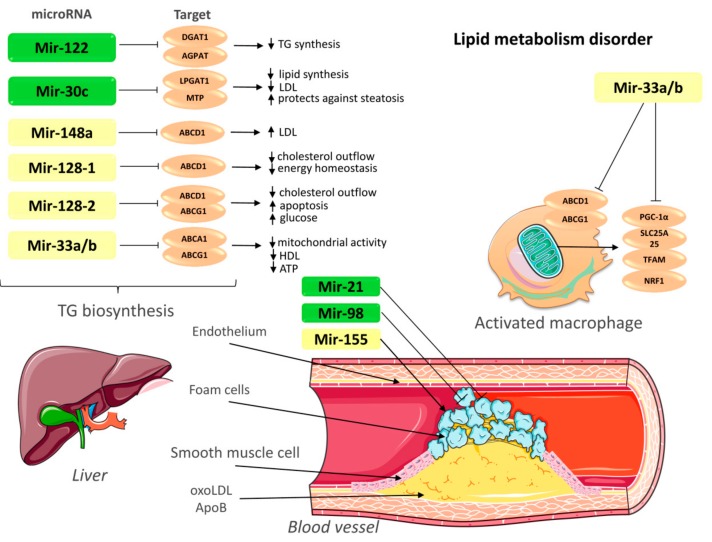
MicroRNAs effect on cellular mechanisms of atherosclerosis. Note: activation →; suppression 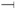; upregulation ↑; downregulation ↓. TG—triglycerides; LDL—low-density lipoprotein; ABCA1—ATP-binding cassette A1; ABCG1—ATP-binding cassette subfamily G member 1; LPGAT1—lysophosphatidylglycerol acyltransferase-1; MTP—Microsomal triglyceride transfer protein; AGPAT—1-acylglycerol-3-phosphate-O-acyltransferase; DGAT1—Diacylglycerol O-acyltransferase 1; PGC-1α—peroxisome proliferator-activated receptor gamma coactivator 1-alpha; SLC25A25—Solute Carrier Family 25 Member 25; NRF1—nuclear respiratory factor; TFAM: transcription factor A, mitochondrial; OxoLDL—oxidized low-density lipoprotein; ApoB—apolipoprotein B. This figure has been created by modifying the templates from Servier Medical Art (https://smart.servier.com).

Studies in mice have shown that overexpression of miR-30c inhibits microsomal triglyceride transfer protein by reducing VLDL production (Figure 1) [21]. The accumulation of LDL leads to the activation of endothelial cells [21]. The study of Sodi R. et al. [22] showed a positive correlation of miR-30c expression with total cholesterol and LDL in people with hypercholesterolemia. MiR-30c inhibits lipid synthesis in the liver by acting on lysophosphatidylglycerol acyltransferase-1 (LPGAT1), an enzyme involved in the synthesis of phospholipids [23]. Perhaps, increased expression of miR-30c is a compensatory mechanism aimed at reducing lipid secretion in hypercholesterolemia. Thus, the increased expression of miR-30c can be used in the treatment of hyperlipidemia and atherosclerosis.

MiR-33 inhibits a cluster of genes that control cellular energy metabolism and cholesterol outflow from macrophages (Figure 1) [24]. The miR-33 family consists of miR-33a and miR-33b encoded in introns of the genes sterol regulatory element-binding proteins 1 (*SREBP1*) and sterol regulatory element-binding proteins 2 (*SREBP2*) [24]. Both miR-33 isoforms have the same sequence, but in the 3′ region, there are differences of two nucleotides [25]. The role of miR-33 in sterol metabolism was originally identified as a regulator of cholesterol transporter expression (*ABCA1* and *ABCG1*) in hepatocytes and macrophages in mice [26,27]. ATP-binding cassette A 1 (*ABCA1*) is the main regulator of reverse cholesterol transport [28]. ATP-binding cassette subfamily G member 1 (*ABCG1*) is involved in the transport of cholesterol and phospholipids in macrophages and can regulate cellular lipid homeostasis in other types of cells [29].

Overexpression of miR-33 suppresses the expression of *ABCA1* and *ABCG1* genes in the liver and contributes to a decrease in the level of high-density lipoprotein (HDL) in the blood plasma of mice (Figure 1) [26]. However, a decrease in miR-33 expression using antisense oligonucleotides (ASO-33) leads to an increase in the expression of the *ABCA1/ABCG1* genes and plasma HDL levels [26]. In addition, the inhibition of miR-33 increased mitochondrial respiration and ATP production by activating miR-33 target genes, such as peroxisome proliferator-activated receptor gamma coactivator 1-alpha (*PGC1-α*) and pyruvate dehydrogenase lipoamide kinase isozyme 4 (*PDK4*) [24].

The introduction of miR-33 antagonists coordinates a network of metabolic processes that increase ATP-dependent cholesterol outflow and contribute to antiatherogenic effects in macrophages [30], emphasizing a new therapeutic pathway to stimulate cholesterol outflow and reduce signs of atherosclerosis, through miR-33.

An increase in circulating apoB-containing lipoproteins (VLDL and LDL) leads to infiltration and retention of these lipoproteins in the arterial wall, which is a critical event in the development of atherosclerosis [16]. MiR-122 is highly expressed in the liver and involved in fatty acid metabolism [10]. Inhibition of miR-122 expression contributes to lower plasma cholesterol and triglyceride levels in animals, and elevated miR-122 levels have been reported in patients with non-alcoholic fatty liver disease, obesity, and type 2 diabetes [31,32]. MiR-122 influences lipid metabolism, making it a promising biomarker for the development of cardiovascular and metabolic disorders. Statins reduce miR-122 levels in the blood circulation, while other types of drugs, such as platelet inhibitors, do not affect its expression [33]. However, the molecular mechanisms and mRNA targets that mediate this effect remain unknown. Wang Y. et al. [34] proved that serum miR-122 can be used as a biomarker for noninvasive diagnosis of atherosclerosis and assessment of the degree of atherosclerotic lesions in the artery. The influence on miR-122 can be used for therapeutic intervention in lipid metabolism in patients with dyslipidemia.

Another potential therapeutic target may be miR-148a and miR-128-1. miR-148a and miR-128-1 control lipoprotein metabolism in the blood by directly affecting the 3′ UTR of the LDL receptors and *ABCA1* genes (Figure 1). MiR-148a may control an extensive network of lipid metabolism regulators, including LDL [28]. Inhibition of miR-148a increases the expression of LDLR in the liver and decreases plasma LDL-C in mice (Figure 1) [28]. MiR-148a is also expressed in adipose tissue and hematopoietic cells [35]. Genome-wide association studies (GWAS) revealed that SNP in the miR-148a locus are associated with obesity [36]. In humans, miR-128 is encoded in the intron of the R3H domain containing 1 gene (*R3HDM1*) on chromosome 2 and co-expressed with its host gene in many tissues [35]. Some authors have established that miR-128-1 plays a key role in the regulation of lipid cholesterol and energy homeostasis of both the proapoptotic molecule and the cholesterol homeostasis regulator [37]. Activation of miR-128-2 reduces cholesterol efflux by inhibiting the activity of the *ABCA1*, *ABCG1* and retinoid X receptor alpha (*RXRα*) genes in human cell lines [37]. Antagonists miR-148a and miR-128-1 also improve the clearance of glucose and increase the sensitivity of the liver to insulin. In addition to regulating lipoprotein metabolism, miR-128-1 regulates *ABCA1* expression in macrophages and improves cholesterol outflow from them [35]. These studies demonstrate that antagonism of miR-148 and miR-128-1 may be a promising therapeutic approach for the treatment of dyslipidemia, atherosclerosis, obesity, and CVDs.

MiR-148a, along with its participation in lipid metabolism, together with DNA methyltransferase 1 (*DNMT1*), regulates the expression of genes involved in the pathogenesis of atherosclerosis [38]. *DNMT1* is a target gene for miR-148a/152. Overexpression of miR-148a/152 leads to suppression of the expression of *DNMT1*, and suppression of miR-148a/152 contributes to increased expression of *DNMT1* [38]. Mutual regulation between miR-148a/152 and *DNMT1* in foam cells probably plays a critical role in the pathogenesis of atherosclerosis, which underlines the potential of its use in therapy.

Thus, lipoprotein metabolism is an important therapeutic target for the treatment of atherosclerosis. Increasing the expression of miR-30c and inhibiting the expression of miR-33, miR-122, miR-128-1, miR-128-2 and miR-148 can be used to treat lipid metabolism disorders and atherosclerosis (Figure 1).

Currently, patents on the use of miR-33 inhibitors (US8859519B2) [39] and mir-27b and mir-148a (WO2014201301A1) [40] mir-128 (WO2012097261A2) [41] for the treatment of dyslipidemia have been created. To develop preclinical models of atherosclerosis therapy, some miRNAs are currently under investigation, such as anti-miR-148a, anti-miR-122, anti-miR-33, anti-miR-92a, anti-miR-33, and anti-miR155 [42].

### 2.2. Technologies for miRNA Delivery

There are two ways to use miRNA for therapeutic interventions. Exogenous miRNAs can be used to replace endogenously expressed miRNAs; on the contrary, oligonucleotides or low molecular weight antagonists can be used to reduce the regulatory effect of natural miRNA genes. The latter approach allows the use of synthetic oligonucleotides to enhance gene expression instead of silencing, which is achieved using antisense and siRNA [43].

There are two main approaches that are considered therapeutic targets for miRNAs: ASOs, including inhibitors, miR sponges and target site blockers (TSB), and miRNA mimics.

MiRNA mimics are RNA molecules that mimic endogenous molecules and help enhance their function. The goal of this approach is to reintroduce miRNA, the expression of which is reduced in the pathological process. MiRNAs are delivered to cells via nanoparticles, encapsulation in liposomes, or miRNA expression vectors [44].

Antagonists of miRNAs are used to inhibit endogenous miRNAs that demonstrate enhanced function in a pathological context (Table 1). These treatments are similar to methods using siRNA. The miRNA antagonist binds to mature miRNA targets with strong affinity, after which the duplex thus formed is destroyed. Since miRNAs can regulate the expression of several genes, inhibition of miRNAs can lead to many side effects. Target site blockers are antisense oligonucleotides designed to bind to a 3′ UTR region complementary to miRNA. Recent developments with miRNAs have accelerated the development of methods and chemical modifications that can stably inhibit miRNAs and optimize their delivery. These techniques are blocked nucleic acids (LNA), peptide nucleic acids (PNA), phosphorothioate groups (phosphorothioate oligonucleotide), miRNA sponges and nanoparticles [45,46].

The base constituting the LNA is a nucleic acid analog in which the ribose ring is chemically modified by the introduction of a methylene bridge. This chemical modification provides the molecule with greater thermodynamic stability and prevents its destruction by nucleases, enhancing its affinity for its target [47]. The effective method of suppressing miRNA functions is called miRNA sponges. This method directly adsorbs miRNAs such that miRNA molecules cannot further bind to their natural mRNA targets [45]. This technology will improve the understanding of the functions of miRNAs and can be used clinically for the treatment of atherosclerosis associated with dysregulation of miRNAs.

The sponge method offers several advantages in the convenience of using and fine-tuning the expression of miRNA [48], which is difficult to achieve by other methods. Sponges have advantages over chemically modified antisense oligonucleotides. Antisense inhibitors are specific for one miRNA, they depend on the extensive complementarity of sequences beyond the seed region [48]. Delivery of a mixture of complementary oligonucleotides may be needed to neutralize the miRNAs family. In addition, many cells are resistant to oligonucleotide uptake and require the use of special delivery vectors, which makes the use of sponges another advantage [48].

The main route of administration of oligonucleotides for systemic use is intravenous or subcutaneous injection. After systemic administration, single-stranded phosphorothioate-modified antisense oligonucleotides are rapidly transferred from the blood to the tissues. The uptake of oligonucleotides by cells is predominantly mediated by endocytosis. Thus, developments with respect to miRNAs enable the stable inhibition of miRNAs and the optimization of their delivery.

### 2.3. Therapeutic Approaches Based on the Use of siRNAs Aimed at the Change in Lipid Metabolism

Currently, the technology of using siRNAs as therapeutic agents is ahead of the technology regarding miRNAs [48]. The siRNAs are highly specific because they affect a single gene. In contrast, miRNAs can target several related genes, often in the same cellular pathway or process, which is more universal to achieving the best therapeutic effect.

siRNAs in the protein complex are responsible for the specific cutting of the target RNA because they are completely complementary (Table 1) [46]. SiRNA RNA differs from miRNA in its origin. For example, miRNAs are encoded by their own genes and cut out from the hairpin formed by the precursor. siRNAs do not have their own genes, representing fragments of longer RNA [49].

There are two different approach for the treatment of atherosclerosis using miRNA: increasing miRNA levels by overexpressing them using synthetic oligoribonucleotides (ORN) or targeted inhibition of miRNA using single-chain antisense oligonucleotides (anti-miRs) [50].

An example of the use of siRNA for therapeutic purposes is the drug Inclisiran. This drug is a chemically synthesized siRNA molecule for sustained specific silencing of the RNA transcription factor of proprotein convertase subtilisin/kexin type 9 (PCSK9) in hepatocytes. PCSK9 contributes to the degradation of the LDL receptor to control plasma LDL cholesterol levels [51]. Thus, PCSK9 causes a steady decrease in the level of low-density lipoprotein cholesterol (LDL) in patients with a high risk of CVDs [52]. The study proved the safety and efficacy of Inclisiran for lowering LDL cholesterol [52].

A study of circulating miRNA profiles in patients with various stages of atherosclerosis is registered on ClinicalTrials.gov (NCT03855891) [53]. Several siRNA-based drugs have passed clinical trials with positive results and are promising treatments for CVDs [54]. Clinical trials are currently underway to evaluate the effectiveness and safety of Inclisiran (ORION-4, NCT03705234 [52]; ORION-10, NCT03399370) [55].

### 2.4. Technologies for siRNA Delivery

Therapeutic approaches based on the use of siRNA include the introduction of synthetic siRNA into target cells to induce RNA interference (RNAi), thereby inhibiting the expression of specific messenger RNA (mRNA) to create the effect of silencing genes [56]. SiRNA and miRNA have similar physicochemical properties but different gene regulation mechanisms: for siRNA, this mechanism is endonucleolytic cleavage of the mRNA, and for miRNA, the mechanism is repression of translation and mRNA degradation [56].

At present, the most common method of delivery is the use of lipid-based nanocarriers in which RNAi agents, such as siRNA and miRNA, are packaged. This method allows the agent to cross the cell membrane [57,58]. In future research, miRNA may be blocked using siRNA to trigger the suppression of the synthesis of many proteins [56].

The success of new therapies using small RNAs depends on the efficiency of the delivery of these molecules to target cells. The method of delivery of miRNAs requires research, because although they are called small, they are still too large to enter into human cells on their own. In addition, while circulating in the bloodstream, these unstable molecules are rapidly destroyed.

However, modern strategies for the treatment of atherosclerosis have disadvantages, since some miRNAs can cause side effects. The use of some miRNAs can cause dyslipidemia, obesity, liver steatosis, and hepatocellular carcinoma, which contributes to the success of such therapy. Further research is needed to develop ways to prevent complications in the treatment of siRNA and miRNA.

## 3. Epigenetic Regulation in the Treatment of Atherosclerosis

**Figure 2:**
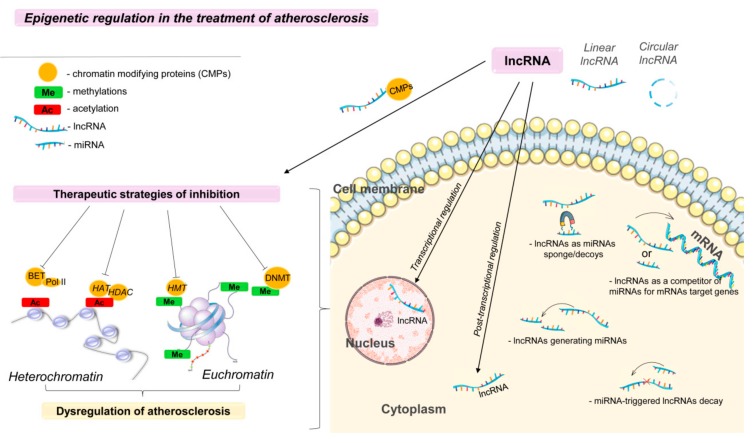
Epigenetic regulation in the treatment of atherosclerosis. Ac—acetylation; Bet—bromodomain and extraterminal protein; Pol II—Polymerase II; HMT—histone methyltransferases; HDAC—histone deacetylase; DNMT:—DNA methyltransferase; HATs—histone acetyltransferases. This figure has been created by modifying the templates from Servier Medical Art (https://smart.servier.com).

In the last decade, increasing evidence has helped to characterize the role of abnormal epigenetic modulation in the development of cardiovascular diseases. Differential DNA methylation profiles are observed in patients with cardiovascular diseases in tissues and cells (including aortic lesions, vascular endothelium, and monocytes) [59], histone methylation and acetylation, which indicates the therapeutic potential for the regulation of epigenetic processes (Figure 2).

Methods based on epigenetic regulation are more manageable for modification or targeted treatment [60]. Epigenetic modifications can be divided into: (1) noncoding RNA mechanisms, such as miRNAs and lncRNAs; (2) DNA methylation and the resulting RNA methylation; (3) histone modifications, including methylation, acetylation, phosphorylation, ubiquitination, ADP ribosylation, and sumoylation [61]. Preparations that act on epigenetic targets can be divided into three main groups. Enzymes that catalyze the addition of a functional group to a protein or nucleic acid; and macromolecules that function as units of recognition, which can distinguish the native macromolecule from the modified; and enzymes that help in removing chemical modifications. Currently, several epigenetic drugs have already received regulatory approval, and many other candidates are undergoing clinical trials [62].

Posttranslational modifications can regulate gene expression by altering chromatin structure. Chromatin can undergo a remodeling process, moving from a tightly packed condensed state (heterochromatin) to an open conformational state (euchromatin), which allows nuclear transcription factors or DNA-binding proteins to access DNA and thus change gene expression (Figure 2) [63]. These modifications include DNA methylation (Me) and methylation and acetylation (Ac) of histone tails. The addition of methyl groups to regulatory regions, such as promoters, using DNA methyltransferase (DNMT) leads to the formation of heterochromatin, which suppresses transcription, preventing the binding of transcriptional complexes with the promoter of the gene [63].

DNA methylation is the first epigenetic modification to be described. This process occurs by adding a methyl group to the C5 position of cytosine in CpG dinucleotides and is regulated by DNMT [64]. Studies have shown that hypomethylation and DNA hypermethylation are characteristic states for atherosclerosis [65]. These profiles affect genes and pathways that regulate the functioning of the endothelium and smooth muscle cells in the pathogenesis of atherosclerosis [64]. A study on the DNA methylation of CpG sites registered a low level of methylation in at Long Interspersed Nuclear Elements (LINE-1) and a high level of methylation of Alu-repeats. [66], which indicates the regulatory role of these processes in the mechanism of atherosclerosis. In addition, a recent epigenome-wide association study (EWAS) of the Japanese population showed that 10 CpG sites were hypermethylated and 16 were hypomethylated in genomic DNA from aortic intima affected by plaque in atherosclerosis, compared to control intima without plaque. A significant contribution is made by DNMT1 and DNMT3a, which alter DNA methylation of Kruppel-like factors 3 (KLF3) and 4 (KLF4) and the homeobox A5 protein, CD31 (cluster of differentiation 31), CDH5 (also known as VE-cadherin) and vWF (von Willebrand factor).

DNMT1 contributes to the progression of atherosclerosis [67]. Hydralazine, a non-nucleoside DNMT inhibitor, has proinflammatory properties and has been approved by the Food and Drug Administration of the USA (FDA) as an antihypertensive drug [68]. Administration of hydralazine suppressed angiotensin II (Ang II) fibrosis and decreased infiltration with inflammatory Mac-2 (+) cells and expression of proinflammatory cytokines, such as interleukin 1b (IL-1b) and IL-6 [68]. Hydrazine has antihypertensive and demethylation activity. Therefore, this molecule is a potential drug for the treatment of atherosclerosis in addition to hydralazine

RG108 is a non-nucleoside inhibitor that directly binds to the active site of DNMT1 and inhibits its activity [69]. Inhibition of the enzyme RG108 can be considered a potential strategy for the prevention or treatment of cardiovascular diseases [69]. Moreover, RG108 activity is associated with a lower level of toxicity [70]. In addition, RG108 showed an inhibitory effect on another DNA methyltransferase 3a (DNMT3A), which is associated with coronary heart disease [71]. Today, a search for DNMT inhibitors is underway. The de novo design technique is currently used for DNMT inhibitors [62]. There is a growing interest in the scientific community to identify and develop small molecules that can be used as epi-drugs targeting DNMT.

In addition to synthetic inhibitors of DNMT, natural, food-derived inhibitors of DNA methylation have been studied (Figure 2) [72]. Modulation of the epigenetic mechanisms of CVD can be achieved by administering epigenetically active agents, such as resveratrol. Resveratrol has a wide range of functions, such as cardioprotective, atheroprotective, and vascular protective activity [73]. Studies have shown that regular consumption of resveratrol is beneficial for the treatment or prevention of various cardiovascular and metabolic diseases [74]. Most clinical and preclinical studies have demonstrated the effectiveness of resveratrol in its administration over short periods [75]. However, further studies are needed to identify the optimal dose of resveratrol and to investigate its long-term effects [72].

DNA demethylation is an opposing mechanism for the reactivation of silenced genes induced by DNMT. DNA demethylation can be catalyzed by members of the TET-methylcytosine dioxygenase family, including TET1, TET2, and TET3, which convert 5-methylcytosine (5-mC) to 5-hydroxymethylcytosine (5-hmC). TET proteins also oxidize 5-hmC to 5-formylcytosine (5-fC) and 5-carboxylcytosine (5-caC) [76]. Increased TET1 expression was observed in neglected atherosclerotic lesions of the carotid arteries compared with healthy arteries [76,77]. On the other hand, it has been described that TET3 is crucial for efficient DNA repair and maintaining genome stability [78]. However, in therapy, effects on TET proteins are limited due to lack of knowledge about them. Recent evidence suggests that TET2 is associated with the phenotypic transformation of vascular smooth muscle cells, endothelial dysfunction, and inflammation of macrophages, which are key factors in atherosclerosis. Therefore, TET2 may still be a potential target for the treatment of atherosclerosis [78].

Targeting DNA methylation/demethylation is a promising way to treat atherosclerosis, similar to the current clinical use of DNA hypomethylating agents for leukemia [79]. The acetylation and methylation of histones in the regulation of inflammation and the development of CVDs has been well-studied [80]. In most cases, treatment methods are aimed only at modifying histones but do not affect the genetic component in the cells. Histone acetyltransferase (HAT) and histone deacetylase (HDAC) are the main enzymes that play an important role in determining the state of histone acetylation [80]. Post-translational histone methyltransferases (HMTs), histone demethylases (HDM), histone deacetylases (HDACs) and histone acetyltransferases (HATs) can also affect gene expression, depending on the site and number of modifications (Figure 2) [59].

Any particular modification of histones is not unique to a particular disease, since it regulates many metabolic pathways. Drugs aimed at a specific modification of histones can be used to treat other diseases. Several HDAC inhibitors are used in the clinic as standards for treatment. There are synthetic inhibitors of HDAC that inhibit HDACs of various classes (I, II, and IV bind to Zn^2+^-containing domains, and III binds to NAD^+^-dependent enzymes) [81].

Bromodomain and extra-terminal (BET) proteins regulate the transcription of lipoproteins and some inflammatory pathways involved in atherosclerosis [82]. RVX 208 (apabetalon) is a new and unique BET protein inhibitor available for the treatment of atherosclerosis. RVX 208 is an oral inhibitor of the BET protein, which increases the transcription of apo A-I, the major HDL receptor [83]. RVX 208 also has some anti-inflammatory properties. RVX 208 may help to prevent the development of type 2 diabetes [83]. RVX 208 increases the production of apo A-I in the liver and intestines, thereby increasing the level of apo A-I in plasma [83]. Apo A-I plasma transports more free cholesterol and phospholipids from peripheral cells and assists in the maturation of HDL [84]. Finally, HDL cholesterol esters are transported to the liver and thus promote cholesterol reverse transport. RVX 208 enhances cholesterol reverse transport and inhibits new inflammatory pathways associated with atherosclerosis [65].

However, phase I and phase II trials showed that the action of the inhibitors is short-lived. In addition, there is no evidence of the inhibitors’ protective action against the development of cardiovascular pathologies [83]. A phase III trial will establish the relative risk reduction in major adverse cardiac events (MACEs) (myocardial infarction (MI) and stroke) (Resverlogix Corp).

The abundance of bromodomain-containing DNA-binding proteins makes the development of substrate-specific drugs extremely difficult. Bromodomain (BRD) recognize only acetylation motifs [85]. The structural features of BRDs contrast sharply with other reader epigenetic domains, such as for example chromodomains [86]. Reading acetylation labels using BRD allows you to regulate the expression of broad-spectrum genes. Either BRD proteins act as scaffolds that recruit and bind large protein complexes, or they act as transcription factors and coregulators themselves. In addition, BRD proteins can contain several catalytic domains, allowing them to act as methyltransferases, ATP-dependent chromatin remodeling complexes or HAT and helicases [62,87].

Thus, drugs that act on epigenetic targets can be divided into three main groups—enzymes that catalyze the addition of a functional group to a protein or nucleic acid; and macromolecules that function as units of recognition, which can distinguish the native macromolecule from the modified; and enzymes that help in removing chemical modifications. Currently, several epigenetic drugs have already received regulatory approval, and many other candidates are undergoing clinical trials [62].

There are several potential candidates for developing the therapy. The complex interaction between addition (e.g., HATs), removal (e.g., HDACs) and recognition containing bromodomain provide a finely tuned pathway of gene regulation. Enhancer of zeste homolog 2 (EZH2), a catalytic subunit of PRC2 (polycomb repressive complex 2), is one of the well-studied HMTs in cardiovascular development and diseases. EZH2 controls LPS-induced macrophage activation and inflammatory responses [88]. Currently, genetic ablation (EZH2 deficiency) or pharmacological inhibition (GSK126) is used to suppress the function of EZH2 methyltransferase [89]. EZH2 reduces the expression of a multitude of pro-inflammatory genes (*IL-6*, tumor necrosis factor-alpha (*TNFα*)), and monocyte chemoattractant protein 1 (*MCP1*) in macrophages. EZH2 is a promising therapeutic target for the treatment of atherosclerosis. In light of the important role of plaque angiogenesis, determining plaque vulnerability [90], further studies are needed to assess the potential role of EZH2 in regulating plaque angiogenesis.

Histone deacetylases processes are also important in the pathogenesis of atherosclerosis. Trichostatin A is a non-specific inhibitor of HDAC class I and class II. Although it has been found to prevent inflammatory markers such as IL-1b and IL-6 and has significant antitumor properties, it appears to play a more detrimental role in atherosclerosis. Treatment with trichostatin A has been found to stimulate proatherogenic markers such as TNF-α, scavenger receptor class A (SRA), CD36, endothelial nitric oxide synthase (eNOS), and vascular cell adhesion molecule -1 (VCAM-1) [67].

The pro-inflammatory role of Jumonji domain-containing protein 3 (JMJD3) has been confirmed for a promising target for the treatment of atherosclerosis [91]. JMJD3 is a specific H3K27me3-demethylase, which increases when LPS is stimulated via the NF-κB-dependent pathway [92]. Using a high-throughput RNA sequencing approach, Neele A. et al. [91] recently observed a decrease in the expression of profibrotic genes in foam cells derived from macrophages in JMJD3 deficient cells, indicating a significant role for JMJD3 in regulating the profibrotic transcriptome in foam cells.

However, there is no detailed information on clinical trials using HDAC inhibitors to treat atherosclerosis in humans [77]. More research is needed to emphasize the specific role of each HDAC and their effect on different cell types during the progression of atherosclerosis. The end result of such studies should justify the need for a comprehensive evaluation of HDAC inhibitors through clinical trials that are currently lacking.

Epigenetic modifications, such as DNA methylation and posttranslational modifications of histones, are promising for the treatment of many diseases and are the focus of epigenetic therapy (Figure 2). The development of treatment strategies aimed at de novo epigenetic changes in various diseases, including atherosclerosis, becomes possible with the emergence of new methods of sequencing, mass spectrometry, chromatin immunoprecipitation, and microchips, and their extensive use contributes to significant progress in scientific research.

There are many potential approaches to the discovery of low-molecular drugs for epigenetic regulation, and each of them must be evaluated for its relevance to a specific goal in the treatment of atherosclerosis. In addition to finding new therapeutic strategies, identifying new epigenetic targets and using them as new treatments for atherosclerosis is a potentially safe and cost-effective way to discover new cardiovascular drugs. Cocktail therapy using drugs such as DNMTi, EZH2i, BETi and HDACi (or tested in clinical trials) for treating cancer is used [93]. However, there are currently very few reports of epigenetic cocktail therapy for the treatment of cardiovascular diseases.

Thus, the atherosclerosis hypothesis, based on epigenetics, has helped to elucidate the molecular mechanisms of the disease, which are traditionally regarded as a chronic inflammatory and lipid disorders of a hereditary nature. Advances in cardiovascular epigenetics, especially studies of associations throughout the epigenome, help to determine the complex interactions of epigenetics in the regulation of lipid metabolism, inflammation and redox status in atherosclerosis.

## 4. Therapeutic Approaches Based on the Use of Long Noncoding RNA Aimed at Atherosclerosis

Another class of noncoding RNAs that have high therapeutic potential is lncRNAs, which are responsible for regulating gene expression from the start of transcription to protein translation [94,95]. Recent studies have indicated that miRNAs, along with lncRNAs, are involved in both DNA methylation and various histone modifications [96]. MiRNA noncoding RNAs and various types of lncRNAs, forming complex molecular networks within the cell, closely interact with each other to regulate the processes of cellular homeostasis [97].

**Figure 3:**
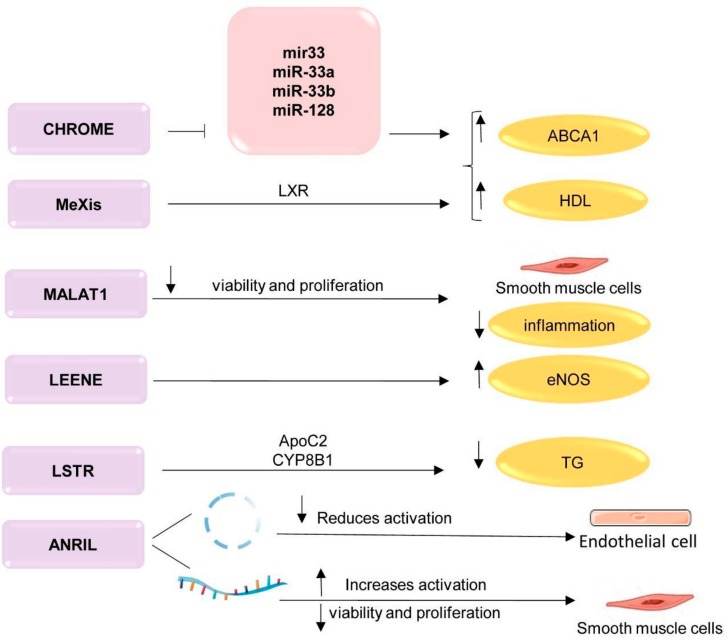
LncRNA and their targets that regulate various processes of atherosclerosis. Activation →; suppression 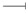; upregulation ↑; downregulation ↓. TG—triglycerides; LXR—liver receptors; ApoC2—apolipoprotein C2; CYP8B1—cytochrome P450 family 8 subfamily B, polypeptide 1; ABCA1—ATP-binding cassette A; HDL—high-density lipoprotein; CHROME—cholesterol homeostasis regulator of miRNA expression; MeXis—Macrophage-expressed LXR-induced sequence; MALAT1—metastasis associated lung adenocarcinoma transcript 1; LSTR—liver specific hepatic triglyceride regulator; ANRIL antisense non-coding RNA in the INK4 locus. This figure has been created by modifying the templates from Servier Medical Art (https://smart.servier.com).

LncRNAs, which coordinate many epigenetic regulatory processes, including the chromatin dynamics, DNA methylation, mRNA stability and noncoding RNAs, and the presence of an epigenetic substrate, possess a therapeutic potential (Figure 2 and Figure 3) [96,98]. In particular, nuclear lncRNAs mainly act on transcription, while cytoplasmic lncRNAs modulate posttranscriptional gene expression [96]. In the nucleus, lncRNAs control the epigenetic state of certain genes, participate in transcriptional regulation, alternative splicing, and form subnuclear compartments [99].

The lncRNA group also includes cyclic forms (Figure 2), which are transcribed in various organisms and from different genomic regions and have diverse biogenesis [100]. LncRNAs are classified according to the region of the genome from which they are synthesized. Scientists have proposed to classify lncRNAs as intergenic, intragenic and overlapping with genes [101]. One of the roles of cyclic RNAs may be their interaction with miRNAs. Cyclic RNAs are not susceptible to exonucleases [102] and can more effectively perform the function of endogenous competitive RNA [103]. These transcripts compete with mRNA for MiRNA binding and reduce the detrimental effect of miRNA on transcriptional and posttranscriptional regulation of gene expression [97]. LncRNAs can be such transcripts in mammals.

LncRNAs are actively involved in the regulation of gene expression [104]. This regulation can be carried out in the cis or trans position of the gene [105] lncRNA is often involved in the regulation with the help of protein factors. LncRNAs direct chromatin-modifying proteins, which can activate or, in the case of tritorax, suppress the gene expression at the level of epigenetic modification of histones or DNA [106].

The interaction between lncRNAs and miRNAs can be classified into (Figure 2):

(a) miRNA-triggered lncRNA decay;
(b) lncRNAs as miRNA sponges/decoys;
(c) lncRNAs as competitors of miRNAs for mRNAs of target genes;
(d) lncRNAs generating miRNAs [107].

Thus, miRNAs and lncRNAs, acting alone or together, control the gene expression through various posttranscriptional mechanisms, thus contributing to reliable regulation of expressed proteins.

The CHROME (cholesterol homeostasis regulator of miRNA expression) lncRNA is a lncRNA that regulates systemic cholesterol homeostasis in the liver and macrophages by inhibiting miRNAs such as miR-33 (Figure 3) [108]. The knockdown of CHROME in human hepatocytes and macrophages increases the miR-27b, miR-33a, miR-33b and miR-128 levels. As a result, the expression of their overlapping target gene networks and their associated biological functions are reduced. In particular, cells without CHROME showed a reduced expression of *ABCA1*, which regulates cholesterol outflow and the formation of nascent HDL particles. Thus, CHROME is one of the key noncoding RNAs that control cholesterol homeostasis in humans, and it can have protective properties against atherosclerosis [108].

Monkeys fed cholesterol-enriched foods had a higher level of CHROME in the liver than monkeys fed a low-fat diet. CHROME expression also increased in cultured human hepatocytes and macrophages in response to cholesterol overload or after treatment with an LXR receptor agonist. Knockdown of CHROME in human hepatocytes and macrophages reduced cholesterol outflow by 50% and reduced protein expression by ATP-binding cassette subfamily A member 1 (*ABCA1*) [109].

The lncRNA MeXis (Macrophage-expressed LXR-induced sequence) is an enhancer of the *ABCA1* gene and interacts with liver receptors (LXRs) (Figure 3). LXR Liver receptors are activated by sterol transcription factors related to the nuclear receptor superfamily [110], and they play an important role in the pathology of atherosclerosis as key gene regulators involved in cholesterol transport [111]. MeXis interacts with and controls the binding of the transcription coactivator promoter DDX17. Thus, a knockout of MeXis resulted in impaired cholesterol outflow and accelerated atherosclerosis in mice [112]. MeXis enhances the transcription of the *ABCA1* gene in an LXR-dependent manner. LXRs regulate the expression of genes involved in macrophage responses to cholesterol and inflammation [113]. LXR activation supports reverse cholesterol transport by induction of a number of genes, including *Abca1*, which encodes the plasma membrane transporter ABCA1. This ATP-dependent transport is critical for the formation of high-density lipoprotein (HDL) [114]. In mice with a knockout of the MeXis gene, the level of *Abca1* is reduced. In mouse bone marrow cells, the inhibition of MeXis altered the chromosome architecture at the *Abca1* locus, impaired cellular responses to cholesterol overload, and accelerated atherosclerosis. Thus, MeXis regulates the expression of *Abca1* through LXR [112]. An impact on the LXR-MeXis-Abca1 axis can enhance the reverse transport of cholesterol in macrophages. Exposure to MeXis is a potential therapeutic targeting strategy for the regulation of macrophage cholesterol efflux.

LncRNA LSTR (liver specific triglyceride regulator) regulates the plasma triglyceride clearance by apolipoprotein C2 (APOC2) and lipoprotein lipase. Blocking of lncRNA LSTR could reduce triglyceride levels in a mouse model with hyperlipidemia (Figure 3) [115]. LncRNA LSTR regulates the TDP-43/FXR/apoC2-dependent pathway to maintain systemic lipid homeostasis. LncRNA LSTR was the first identified lncRNA in the liver, which was described as a regulator of glucose and lipid metabolism in the liver. Depletion of LSTR in the mouse liver induces an increased clearance of triglycerides in a manner dependent on the increased expression of apolipoprotein C-II (apoC2). LncRNA LSTR directly binds the transactive response (TAR) of DNA-binding protein (Tdp-43) and forms a molecular complex that regulates the expression of hepatic cytochrome P450 (Cyp8b1), which induces the expression of ApoC2. Depletion of LSTR can reduce blood glucose and triglyceride levels in a hyperlipidemic mouse model [115].

The *ANRIL* (antisense non-coding RNA in the INK4 locus) noncoding RNA is a key molecule of atherogenesis, located at the Chr9p21 locus. *ANRIL* affects several cell types related to the development of cardiovascular diseases [116]. *ANRIL* in a cis position leads to high levels of linear *ANRIL* but reduces the annular level of *ANRIL* [116]. The balance of the linear and circular RNA *ANRIL*, defined by the Chr9p21 genotype, regulates the molecular pathways and cellular functions involved in atherogenesis [116]. *ANRIL* reduces the viability and proliferation of smooth muscle cells [117] and activates inflammation and apoptosis in endothelial cells [118]. In addition to acting in the cis-conformation, *ANRIL* acts in the trans-conformation (through Alu elements) to regulate other genes that are involved in proatherogenic pathways (Figure 3) [119].The molecular mechanisms of how the ratio of linear and circular forms of *ANRIL* is controlled by the genotype in the locus is currently unknown. It will be important to determine which gene regulatory elements in the *ANRIL* gene can lead to abnormalities associated with the risk of atherosclerosis [120].

A commonly expressed and evolutionarily conserved lncRNA *MALAT1* (metastasis associated lung adenocarcinoma transcript 1), is less actively expressed in atherosclerotic plaques [121]. Reduced expression of MALAT1 in hematopoietic cells contributes to the development of atherosclerosis and inflammation in mice in vivo [122]. In ApoE ^−/−^ heterozygous mice with MALAT1 deficiency, an increased level of inflammation and the development of atherosclerosis were observed [123].

The knockdown of MALAT1 in vascular smooth muscle cells (VSMCs) and endothelial cells (ECs) led to cell cycle arrest and the reduction in cell proliferation (Figure 3) [124]. In addition, the silencing of MALAT1 with LNA-GapmeR inhibited the proliferation and formation of primary endothelial cells (SMMECs) in vitro [125]. The mechanism of action resulting in the inhibition of proliferation is associated with *Malat1* binding to the vascular endothelial growth factor receptor 2 (VEGFR2) gene [126]. Taken together, these results suggest that *Malat1* may play a critical role in the development of angiogenesis [126].

The long intergenic noncoding RNa-p21 (lincRNA-p21) is reduced in patients with coronary heart disease and in mice with atherosclerosis [127]. LincRNA-p21 regulates the p53-dependent proliferation and apoptosis of smooth muscle cells [127].

Endothelial nitric oxide synthase (eNOS) is an important element of endothelial homeostasis and vascular function [128]. Transcription of eNOS is regulated by KLF2 and KLF4, two key transcription factors [129]. The lncRNA LEENE enhances expression of eNOS [130]. The inhibition of LEENE at the level of transcription suppresses eNOS, while the overexpression of LEENE increases the level of eNOS and bioavailability of NO [130]. In addition to the regulation of eNOS, LEENE can interact with genomic loci encoding certain sets of genes [131]. These genes are involved in multiple pathways, which are critical for endothelial homeostasis, for example, cell adhesion and VEGF signaling [131].

Lyu Q. and co-author [132] found that lncRNAs are regulators of the phenotype of endothelial cells. The mechanisms of action of lncRNA *SENCR* (smooth muscle and endothelial cell enriched migration/differentiation-associated) on various cell types are unknown. An increase in the level of lncRNA *SENCR* in the endothelial cell line after the stress of laminar shift was shown. Knockout *SENCR* led to a violation of the integrity and increased the permeability of the membrane of endothelial cells. Thus, the authors suggest that lncRNA *SENCR* is sensitive to flow-responsive, contributes to the integrity of endothelial cells through physical association with CKAP4, thereby stabilizing CDH5 associated with the cell membrane [132].

Identifying the functions of lncRNAs in atherosclerosis may be the key to identifying mechanisms that contribute to the development of this pathology. Thus, increasing the levels of CHROME, MeXis, MALAT1, lincRNA-p21, or LEENE and inhibiting the activity of LSTR or ANRIL can be used in the treatment of disorders of lipid metabolism and atherosclerosis (Figure 3). In the future, it is necessary to determine whether lncRNAs act synergistically and whether they play redundant and/or compensatory roles with other unregulated lncRNAs and/or mRNA associated with the development of atherosclerosis.

### Technologies for lncRNA Delivery

The most common mechanism of epigenetic regulation is methylation directed at lncRNA. Methylation of histones or DNA in the CpG sequences using methyltransferase 3, histone H3, lysine 9, methyltransferase, and polycomb repressive complex 2 (PRC2) lead to the stable repression of genes [133]. The emergence of RNA-seq and other technologies “-omics” in the last decade has served as a catalyst for the identification of many new lncRNAs. With the growing number of lncRNAs candidates and the improvement of accurate mapping and annotation approaches, it is possible to functionally analyze these areas to develop new strategies for identifying noncoding genomic risk factors.

The manipulation of lncRNAs is based on the introduction of oligonucleotides by injection or inhibition of lncRNA expression [134]. Currently, there are two main methods of suppressing lncRNA expression that are used in preclinical models. The first is the use of RNAi-based methods, such as siRNA and LNA-GapmeR antisense oligonucleotides (ASO), which induce RNAase cleavage [135]. Usually, the RNAi method, which includes siRNA and a short hairpin RNA (shRNA) that can be delivered via a viral vector, is used primarily for lncRNA, which are localized in the cytoplasm [136]. GapmeR can be used for core-localized lncRNA because it induces degradation with RNase-H and is RISC-independent [137]. GapmeR can be used to modulate the expression of lncRNA in vivo [138]. The LNA-GapmeR-mediated silence of MALAT1 in the endothelial cells of skeletal muscle effectively reduces the formation, migration, and proliferation of endothelial cells [99].

Today, the CRISPR/Cas9 system is known as “molecular scissors” and is widely used in various types of research [136]. Genome editing with CRISPR/Cas9 is a new tool for modulating gene expression associated with ncRNA, including the manipulation of lncRNA [135]. CRISPR inhibition (CRISPRi) suppresses the expression of miR-21 [139] and lncRNAs, including GAS5, MALAT1, UCA1, and lncRNA-21A [139].

Research into the involvement of lncRNAs in the pathogenesis of atherosclerosis should be carried out on primary human cells or induced pluripotent stem cell (iPSC) derived vascular cells because lncRNAs are more species specific. In the context of atherosclerosis, genetic manipulation with lncRNAs via antisense oligonucleotides or CRISPR/Cas9 to remove or activate/repress the expression of lncRNA enables the establishment of markers or therapeutic targets for cardiovascular diseases. It is not completely clear how lncRNA interacts with each other and other noncoding RNA, their role is excessive or compensatory. Understanding the versatility of lncRNA may be the key to determining pathways associated with atherosclerosis.

## 5. Drug Delivery Using Nanoparticles

Despite significant progress in creating systems that model atherosclerosis, accurately testing potential epigenetic inhibitors for the treatment of atherosclerosis requires much more research. Delivery of these compounds to the site of injury remains a problem that can be solved by the development of nanomedicine.

Nanoscale drug delivery systems are obtained using various organic, inorganic, lipid, and polymeric biomaterials. Numerous studies have shown that the structural and physico-chemical characteristics of nanoparticles can affect their performance in vitro and in vivo. The large surface to volume ratio facilitates the design of multifunctional nanoparticles, i.e., the shape and surface charge of the nanoparticles. Scientists have shown that the shape and surface charge affect the penetration of nanoparticles via the blood–brain barrier, biodistribution in organs, and cellular absorption. Nanoparticles are classified based on various properties; morphologically, they can be divided into nanospheres, nanotubes, and dendrimers and linear, block, and graft grafted structures (grafts). Nanoparticles can also be categorized, based on the material they are made from, as natural, synthetic, hybrid, or metallic.

Nano drugs have significant potential in the prevention, diagnosis, and treatment of various diseases, including atherosclerosis. It should be noted that the use of nanosystems significantly reduces the risk of side effects. Targeted drug delivery to atherosclerotic foci and plaques will be a much more effective method than classical treatment methods.

Nanoparticles can be used as visualization tools to detect vulnerable atherosclerotic plaques; similar theranostic strategies have already demonstrated the potential for identifying and treating other diseases (including cancer and neurodegenerative disorders) using a variety of imaging methods, including optical imaging, magnetic resonance imaging (MRI), ultrasound and photoacoustics, computed tomography (CT), and nuclear imaging based on single-photon and positron emission tomography [140].

Atherosclerotic molecular markers are the main targets that can be used to develop targeted nanoparticles [141], which include VCAM-1, ICAM-1, P-selectin, E-selectin, and αvβ3-integrin over-expressed on activated endothelium [141]. High-density lipoproteins (HDLs) are responsible for modulating inflammation, and are involved in the reverse transport of cholesterol, therefore, are also potential targets. Intimal macrophages are critical cells in the development of atherosclerosis and can absorb nanoparticles by phagocytosis, so they are potential targets for nanoparticles too [142].

The unique characteristics of nanomaterials (for example, shape, size, and charge) make them promising tools for both diagnostics and therapeutic approaches, but today there are still many limitations and shortcomings in the clinical use of nanoparticles. Currently, out of 51 FDA-approved nano-medicines and 77 products undergoing testing, only six were indicated for atherosclerosis, in particular: (1) Tricor (Lupine Atlantis, 2004) with nanocrystalline fenofibrate, (2) Rapamune sirolimus immunosuppressant (Wyeth Pharmaceuticals, 2000), (3) plasmon immunosuppressant sirolimus (2000), (4) plasmonic immunosuppressant sirolimus (2000), (5) plasmon-containing immunosuppressant sirolimus Rapamune (plastophage). In addition, atherosclerosis is being treated with stem cells (NCT01270139), as well as MRI with iron and enhanced ferumoxytol for the assessment of myocardial infarction (NCT01995799, NCT01323296), including Feridex/Endorem superparamagnetic imagers (superparamagnetic ferric oxide of nanocephanol) dextran-coated (SPION) (AMAG Pharmaceuticals, 1996, 2008) and GastroMARK; Lumirem (SPION, coated with silicone; AMAG Pharmaceuticals, 2001, 2009) [143,144]. However, 98.83% of developments in this area are pre-clinical [145].

Today it is not clear how to control the removal of nanoparticles from the body because they often also accumulate in the organs of the reticuloendothelial system (RES), due to their polydispersity and/or the complex reproducibility of their preparation, or because of the difficulty of their scaling and high production costs, especially when particles are multifunctional [146].

Nanotechnology is known as the industrial revolution of the 21st century, and its medical use, nanomedicine, has made promising achievements, such as drug delivery systems and visualization, among many others.

## 6. Summary and Perspective

Atherosclerotic CVD is one of the key causes of mortality, and to date, there are many methods of correcting specific components of this disease. This review examined several of the newest and most promising approaches in the treatment of atherosclerosis, including some that have already demonstrated their effectiveness in clinical trials. Therapy based on miRNA is a new area, demonstrating significant promise. Studies in mice, primates, and early human trials clearly demonstrate the potential of using miRNAs as valuable therapeutic agents.

In the treatment of atherosclerosis, a number of noncoding RNAs have been identified, such as miRNA, siRNA, and lncRNA, which are responsible for the effects on the basic elements in the pathogenesis of the disease.

Presently, some features of the regulation of gene expression using ncRNAs have been investigated, but their enormous therapeutic potential is already becoming clear. Noncoding RNAs are involved in many epigenetic regulatory processes of targeted genes in atherosclerosis. LncRNA possess therapeutic potential by coordinating many epigenetic regulatory processes, including chromatin dynamics, DNA methylation, and the stability of mRNA and other noncoding RNAs. Epigenetic-based atherosclerosis hypotheses have improved the understanding of the molecular mechanisms of atherosclerosis, which is traditionally regarded as a chronic inflammatory and lipid disorder, with genetic codes being a key determinant.

However, there are several unsolved problems that deserve further study, such as safe and effective delivery methods, the long-term effectiveness of their action, and side effects after long-term treatment. It is still not entirely clear how to achieve the desired specificity of miRNA application, aimed at a specific metabolic pathway. Given the relatively short period of time since the discovery of miRNAs, progress seems to be sufficient to justify optimism regarding the development of new therapeutic agents based on miRNAs.

## Figures and Tables

**Table 1:** The mechanism of action of noncoding RNA.

ncRNA	Impact Targets	Impact Level	Therapeutic Strategies Based on ncRNA	
			Downregulation	Upregulation
**miRNA**	Regulate the expression of several genes	Repression translation degradation of mRNA	Anti-miRNA: Inhibition endogenous miRNA; miR sponges; Target site blocker; CRISPR	Mimics miRNA; re-introduction of miRNA ORN
**siRNA**	Highly specific, complementary to the target gene	Endonucleolytic cleavage of the target mRNA	–	–
**lncRNA**	Regulate the expression of several genes	Regulation of gene expression from the start of transcription to protein translation	Inhibition lncRNA: LNA-GapmeR; Short hairpin RNA CRISPR	–

**Note:** ncRNA—noncoding RNA; ORN—synthetic oligoribonucleotides; siRNA—small interfering RNA; miRNA—microRNA; lncRNA—long noncoding RNA; LNA-GapmeR—Locked Nucleic Acid GapmeR, CRISPR—clustered regularly interspaced short palindromic repeats.
